# Accuracy of breast magnetic resonance imaging in evaluating the response to neoadjuvant chemotherapy: a study of 310 cases at a cancer center

**DOI:** 10.1590/0100-3984.2018.0149

**Published:** 2019

**Authors:** Erika Marina Solla Negrão, Almir Galvão Vieira Bitencourt, Juliana Alves de Souza, Elvira Ferreira Marques

**Affiliations:** 1 Hospital de Câncer de Barretos/Instituto de Prevenção Campinas, Barretos, SP, Brazil.; 2 A.C.Camargo Cancer Center - Departamento de Imagem, São Paulo, SP, Brazil.

**Keywords:** Breast neoplasms, Drug therapy, combination, Magnetic resonance imaging, Triple negative breast neoplasms, Receptor, ErbB-2, Neoplasias da mama, Quimioterapia combinada, Ressonância magnética, Neoplasias de mama triplo negativas, Receptor ErbB-2

## Abstract

**Objective:**

To evaluate the accuracy of magnetic resonance imaging (MRI) of the breasts in the identification of a pathological complete response in patients with breast cancer undergoing neoadjuvant chemotherapy (NAC).

**Materials and Methods:**

This was a single-center, retrospective, observational study designed to validate a diagnostic test. The following variables were evaluated: age; results of the histological and immunohistochemical analysis of the biopsy; post-NAC MRI findings; and results of the histological analysis of the surgical specimen, using the residual cancer burden index. The radiological response, as assessed by MRI, was compared with the pathological response, as assessed by histological analysis of the surgical specimen (the gold standard method).

**Results:**

We evaluated 310 tumors in 308 patients. The mean age of the patients was 47 years (range, 27-85 years). For identifying a pathological complete response, breast MRI had an overall accuracy of 79%, with a sensitivity of 75%, specificity of 83%, positive predictive value of 75%, and negative predictive value of 83%. When that accuracy was stratified by molecular subtype, it was best for the HER2 subtype, with a sensitivity and specificity of 82% and 89%, respectively, followed by the triple-negative subtype, with a sensitivity and specificity of 78% and 83%, respectively.

**Conclusion:**

Breast MRI showed good accuracy in the prediction of a pathological complete response after NAC. The sensitivity and positive predictive value were highest for the HER2 and triple-negative subtypes.

## Abstract (pt)

**Objetivo:**

Avaliar a acurácia da ressonância magnética (RM) das mamas na identificação de resposta patológica completa em pacientes com câncer de mama submetidas a quimioterapia neoadjuvante (QTN).

**Materiais e Métodos:**

Teste de validação diagnóstica, realizado por meio de estudo observacional, unicêntrico e retrospectivo. As variáveis avaliadas no estudo foram idade, resultado histológico e imuno-histoquímico da biópsia, análise da RM após QTN e análise histológica da peça cirúrgica, com cálculo do índice *residual cancer burden*. Os resultados da resposta radiológica pela RM foram comparados com a resposta patológica na peça cirúrgica (padrão ouro).

**Resultados:**

Foram incluídos 310 tumores de 308 pacientes com média de idade de 47 anos (variação: 27 a 85 anos). A acurácia da RM foi 79%, com sensibilidade de 75%, especificidade de 83%, valor preditivo positivo de 75% e valor preditivo negativo de 83%. Estratificando-se por subtipo molecular, a detecção da resposta patológica pela RM obteve os melhores porcentuais de acerto no subtipo HER2 superexpresso, com sensibilidade e especificidade de 82% e 89%, respectivamente, seguido do subtipo triplo negativo, com sensibilidade e especificidade de 78% e 83%, respectivamente.

**Conclusão:**

A RM demonstrou boa acurácia na predição de resposta patológica completa após QTN. A sensibilidade e o valor preditivo positivo foram mais altos nos subtipos triplo negativo e HER2 superexpresso.

## INTRODUCTION

Although neoadjuvant chemotherapy (NAC) for breast cancer was initially used only as a salvage therapy for inoperable tumors, it has since made significant progress toward being an accepted treatment in other contexts. It is now more widely used and has been shown to be as effective as is postoperative adjuvant therapy^(^^1^^-^^3^^)^, with potential advantages such as primary tumor shrinkage-possibly leading to conversion from mastectomy to breast-conserving surgery and from axillary lymph node dissection to sentinel lymph node biopsy-as well as eliciting better patient responses, as determined by *in vivo* evaluation and by detection of a pathological complete response (pCR). Achieving a pCR has been proposed as a surrogate endpoint for long-term clinical benefit, given that had greater overall and disease-free survival have been shown to be better in patients who achieve a pCR, which has greater prognostic value in aggressive (triple-negative and HER2+) tumor subtypes^(^^4^^)^.

The evaluation of the response to NAC usually relies on a combination of clinical examination and imaging tests. Magnetic resonance imaging (MRI) has proven to be the most sensitive imaging modality for monitoring patient response to NAC^(^^5^^-^^7^^)^. Enhancement patterns seen on dynamic contrast-enhanced MRI can detect tumor angiogenesis, the accompanying changes in tumor microcirculation, and even increased permeability of the newly formed vessels. Thus, MRI provides insight into the pathophysiology of the tumor response to NAC, allowing an earlier, more accurate assessment than does the purely anatomical evaluation performed with mammography and ultrasound^(^^8^^)^.

Although MRI is an excellent test, it is not perfect. Discrepancies between MRI findings and surgical pathology findings are well documented. Overestimation of residual disease may result in more extensive surgery than actually required, resulting in more extensive breast-conserving surgery, wider surgical margins, and unnecessary mastectomy, whereas underestimation may result in incomplete resection, resulting in positive margins and re-excision^(^^9^^,^^10^^)^. Therefore, it is important to know when MRI findings, especially those indicating a radiological complete response (rCR), are reliable and when they are less accurate^(^^11^^)^. There is evidence that the accuracy of MRI in evaluating the response to NAC is dependent on the tumor subtype; the strongest evidence coming from multicenter trials^(^^12^^)^.

The objective of this study was to evaluate the accuracy of breast MRI in identifying a pCR in patients with breast cancer submitted to NAC.

## MATERIALS AND METHODS

This was a retrospective validation study of a diagnostic test, in which we analyzed imaging findings and electronic medical records. The study was approved by the local research ethics committee. We included patients with a diagnosis of breast cancer who were submitted to NAC and subsequent preoperative breast MRI between October 2014 and July 2017. Patients who did not undergo surgical treatment at our center were excluded, as were those for whom the pathology study was incomplete or provided insufficient data.

The following variables were evaluated: age; histology and immunohistochemistry of the biopsy; post-NAC breast MRI findings; and surgical specimen histology. We used immunohistochemistry, (estrogen and progesterone) hormone receptor biomarkers, HER2, and Ki-67 to classify tumors as follows: luminal A (hormone receptor-positive and Ki-67 < 20%); luminal B (hormone receptor-positive and Ki-67 ≥ 20%); luminal B HER2+ (hormone receptor-positive and HER2-positive); HER2-enriched (hormone receptor-negative and HER2-positive); and triple-negative (negative for all receptors). The surgical resection specimen was analyzed and classified according to the protocol of the pathology department of our center, which includes determining the residual cancer burden (RCB) index^(^^13^^)^. The pathological response was divided into categories by RCB index: class 0 (pCR); class I (minimal residual disease); class II (moderate residual disease); or class III (extensive residual disease). For purposes of comparison with the MRI results, we defined a pCR as resolution of the invasive mammary disease. Exclusively *in situ* residual disease (ductal carcinoma *in situ*) and exclusively axillary micrometastasis were defined as complete responses.

For the acquisition of the MRI images, patients were placed in the prone position in a 1.5 T scanner (Signa HDxt; GE Healthcare, Milwaukee, WI, USA, or Achieva; Philips Medical Systems, Best, The Netherlands) with a dedicated breast coil. Post-NAC MRI images were analyzed by two radiologists with four and ten years of experience in breast radiology, respectively, who worked independently and were blinded to the surgical results, to determine whether an rCR had been achieved or not. The lesions detected in the breasts were classified according to the criteria established by the Breast Imaging Reporting and Data System (BI-RADS) for MRI. To evaluate the response to NAC on MRI, we looked for an area of abnormal enhancement where the lesion had been or where a clip was placed (or susceptibility artifact, when present) in the early phase (approximately 100 s after contrast administration) and in the delayed phase (360 s after contrast administration), in the axial and sagittal planes, respectively. Patients in whom the contrast enhancement of the affected area was equal to or less than that of normal breast tissue were considered to have achieved a complete response, and the MRI was classified as negative for residual disease in those cases. To evaluate the level of agreement or reproducibility of the data analyzed, we calculated the kappa coefficient and stratified the data by the degree of reproducibility, respectively^(^^14^^)^.

**Figure 1:**
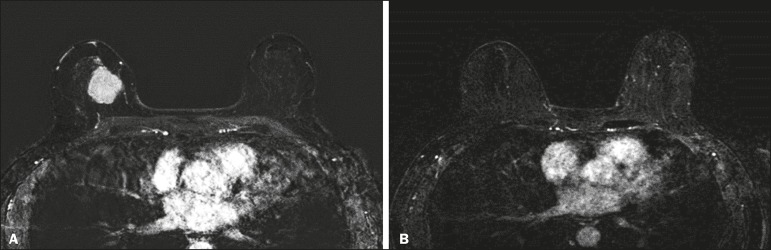
Example of a true-positive result. Pre- and post-NAC MRI (**A** and **B**, respectively) of a 73-year-old patient with invasive ductal carcinoma of no special type, triple-negative subtype, histological grade III, and nuclear grade 3, with a Ki-67 value of 40%. Final pathologic TNM staging: ypT0ypN0.

**Figure 2:**
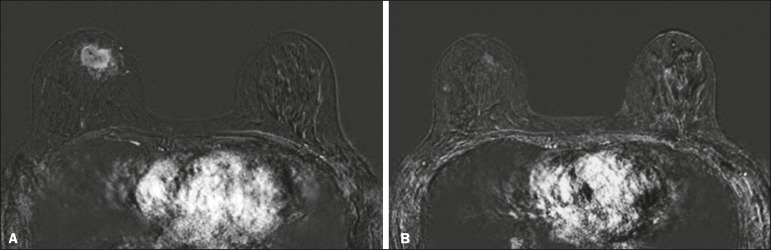
Example of a true-negative result. Pre- and post-NAC MRI (**A** and **B**, respectively) of a 54-year-old patient with invasive ductal carcinoma of no special type, luminal B subtype, histological grade III, and nuclear grade 3, with a Ki-67 value of 40%. Final pathologic TNM staging: ypT2ypN1aypMX.

**Figure 3:**
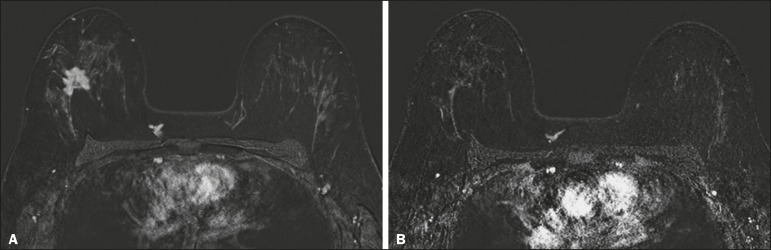
Example of a false-positive result. Pre- and post-NAC MRI (**A** and **B**, respectively) of a 37-year-old patient with invasive ductal carcinoma of no special type, luminal B subtype, histological grade III, and nuclear grade 3, with a Ki-67 value of 30%. Final pathologic TNM staging: ypT1bypN0(sn).

**Figure 4:**
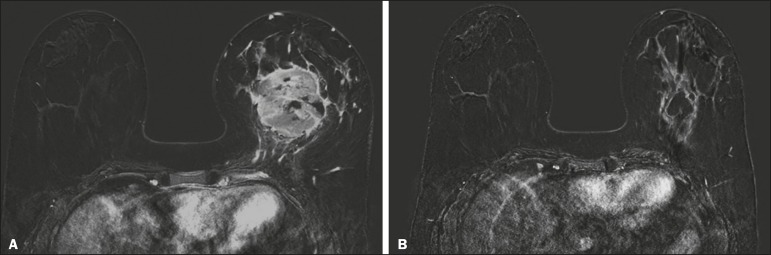
Example of a false-negative case. Pre- and post-NAC MRI (**A** and **B**, respectively) of a 34-year-old patient with invasive ductal carcinoma of no special type, triple-negative subtype, histological grade III, and nuclear grade 3, with a Ki-67 value of 90%. Final pathologic TNM staging: ypT0ypN0.

The radiological response seen on the post-NAC MRI, classified either as rCR or non-rCR, was compared with the surgical specimen pathology (the gold standard method), classified either as pCR or non-pCR. Cases in which an rCR and pCR were achieved (a true-positive result for both) (Figure 1) were considered concordant, as were those in which neither was achieved (a true-negative result for both) (Figure 2), whereas those in which one was achieved and the other was not (an rCR without a pCR; false-positive result) (Figure 3) or a pCR without an rCR; false-negative result) (Figure 4) were considered discordant. We used a pCR as the concept of positivity, and the sensitivity was therefore calculated on the basis of the ratio between the number of true-positive rCR results on MRI and the total number of tests showing a pCR. Likewise, we used failure to achieve a pCR (non-pCR) as the concept of negativity. The specificity was determined by calculating the ratio between the number of true-negative rCR results and the total number of tests showing failure to achieve a pCR. The negative predictive value, positive predictive value, and overall accuracy of MRI were also calculated, the pathological response being considered the gold standard.

## RESULTS

We analyzed 310 tumors in 308 patients (including 2 cases of synchronous tumors). Patient ages ranged from 27 to 85 years, with a mean of 47 years and a median of 46 years. Two hundred and nineteen patients underwent pre-NAC MRI. Among those patients, the predominant finding was a nodular lesion, which was seen in 149 (68%), followed by a non-nodular lesion, which was seen in 27 (12%). Concomitant nodular and non-nodular lesions were seen in 43 cases (20%). The neoplasia presented as a solitary lesion in 137 patients (63%) and as multifocal/multicentric disease in 82 (37%). Using the tumor-node-metastasis (TNM) staging system, we classified 19 cases (9%) as T1c, 139 cases (63%) as T2, and 61 cases (28%) as T3. Atypical ipsilateral axillary lymph nodes considered suspicious were seen in 158 cases (72%).

All cases were classified by molecular subtype. The most common subtype was luminal B, seen in 177 cases (57%), of which 55 (18%) were classified as luminal B HER2+. The triple-negative subtype was seen in 90 cases (29%) whereas the HER2-enriched subtype was seen in 31 cases (10%) and the luminal A subtype was seen in 12 cases (4%).

Post-NAC MRI images showed an rCR in 126 patients (41%) and no rCR in 184 (59%). Among the tests showing residual lesions, those lesions were classified as nodular in 67 cases (22%), as non-nodular enhancements or residual foci in 106 (34%), and as a mix of nodular and non-nodular enhancements in 10 (3%).

The most common surgery after NAC was mastectomy, which was performed in 206 cases (67%), including the two cases of synchronous tumors. Breast-conserving surgery was performed in 102 cases (33%). Mastectomy rates were highest for the luminal subtypes, mastectomy being performed in 102 (76%) of the 134 cases of luminal breast cancer, compared with 55 (39%) of the 141 cases of luminal B HER2+ or HER2-enriched breast cancer and 51 (57%) of the 90 cases of triple-negative breast cancer.

Histological examination of the surgical specimen showed residual neoplasm in 184 (59%) of the 310 cases, no residual neoplasm in 114 (37%), exclusively *in situ* ductal carcinoma in 10 (3%), and axillary lymph node disease only with no residual breast lesion in 2 (0.6%). On the basis of the RCB classification, we categorized 116 cases (37%) as class 0; 35 (11%) as class I; 98 (32%) as class II; and 61 (20%) as class III.

Among the 206 patients submitted to mastectomy, the histological study of the surgical specimen showed no residual lesion in 77 (37%). Of the 102 patients submitted to breast-conserving surgery, 53 (52%) had residual lesions in the surgical specimen.

Among the 310 tumors analyzed, MRI correctly identified the absence of invasive mammary disease, which was later confirmed by the surgical specimen study (true-positive results), in 94 cases (30%) and positive invasive mammary disease, which was later confirmed by the surgical specimen study (true-negative results), in 152 cases (49%). However, MRI incorrectly classified 32 cases as having achieved an rCR, those cases later being found to harbor residual invasive disease (false-positive results), and another 32 as having failed to achieve an rCR, those cases later being found to have achieved a pCR in the surgical specimen study (false-negative results). As a result, in the present study, MRI had 79% accuracy, 75% sensitivity, 83% specificity, a 75% positive predictive value, and an 83% negative predictive value.

MRI detection of the pathological response was best for the HER2-enriched subtype, for which it had 82% sensitivity and 89% specificity, followed by the triple-negative subtype, for which it had 78% sensitivity and 83% specificity (Table 1).

**Table 1:** Accuracy (including sensitivity, specificity, PPV, and NPV) of MRI, by molecular subtype.

Subtype	Sensitivity	Specificity	PPV	NPV	Overall accuracy
All cases (n = 310)	75%	83%	75%	83%	79%
Luminal (n = 132)	58%	85%	48%	89%	79%
Luminal B/HER2 (n = 55)	78%	72%	67%	82%	74%
HER2-enriched (n = 31)	82%	89%	95%	67%	83%
Triple-negative (n = 92)	78%	83%	88%	71%	80%

PPV, positive predictive value; NPV, negative predictive value.

Of the 32 false-positive cases, 25 (78%) were of the luminal subtype-luminal A (n = 2); luminal B (n = 14); and luminal B HER2+ (n = 9)-one (3%) was of the HER2-enriched subtype, and six (20%) were of the triple-negative subtype. Examination of the surgical specimen revealed five false-positive cases with extensive residual disease (RCB class III), all of which were estrogen receptor-positive (four luminal and one luminal B HER2+). Both evaluators characterized those cases as having achieved an rCR. Of the 32 false-positive cases, 14 (42%) were classified as RCB class I and 13 (41%) were classified as RCB class II.

Of the 32 false-negative cases, 16 were of the luminal subtype-luminal B (n = 11); and luminal B HER2+ (n = 5)-four (12%) were of the HER2-enriched subtype, and 12 (37%) were of the triple-negative subtype.

The level of interobserver agreement was considered substantial for all subtypes. Nevertheless, it was highest for the luminal A and luminal B subtypes, as shown in Table 2.

**Table 2:** Interobserver agreement, by molecular subtype.

Subtype	*P*	Kappa	95% CI	Agreement
Luminal	< 0.001	0.780	0.612-0.949	Substantial
Luminal B HER2+ and HER2-enriched	< 0.001	0.652	0.442-0.862	Substantial
Triple-negative	< 0.001	0.626	0.423-0.828	Substantial
All cases	< 0.001	0.713	0.602-0.823	Substantial

95% CI, 95% confidence interval.

## DISCUSSION

The results of the present study show that MRI has 79% accuracy in identifying the post-NAC pathological response of patients with breast cancer. Its sensitivity for detecting a pCR was 75%. Its accuracy and sensitivity were highest for the HER2-enriched and triple-negative subtypes.

A recent meta-analysis and systematic review of the literature on MRI detection of a pCR in patients submitted to NAC identified 1560 relevant studies, of which 57 were considered eligible for inclusion^(^^15^^)^. Of those 57 studies, only two evaluated samples of more than 300 patients, one involving 746 and 569 patients, respectively^(^^16^^,^^17^^)^. The remaining studies had sample sizes ranging from 21 to 264 patients. The meta-analysis found that MRI had a combined sensitivity of 64% (range, 56-70%), a pooled specificity of 92% (range, 89-94%), and an accuracy of 88% (range, 85-91%). In the present study, which included 310 cases, MRI had a higher sensitivity (75%), although its specificity and accuracy were slightly lower (83% and 79%, respectively).

Other meta-analyses, carried out with the aim of determining the value of MRI in predicting pCR and found greater variations. One, including 35 studies, found sensitivity values of 25-100% and specificity values of 50-97%^(^^9^^)^. Prior to that, another meta-analysis, including 25 studies, found sensitivity values of 56-70% and specificity values of 90-92%^(^^18^^)^.

Another meta-analysis^(^^19^^)^, evaluating 44 studies that included a collective total of 2050 patients, found that the specificity of MRI was higher when the criterion for establishing an rCR was enhancement equal to or less than that of the normal breast parenchyma-the same criterion used in the present study-than when it was complete absence of contrast enhancement (83% vs. 54%; *p* = 0.02). The use of this criterion can facilitate the planning of breast-conserving surgery. According to the authors of that meta-analysis, a false-positive result for residual malignancy can be attributed to an increase in vascular permeability caused by inflammatory or reactive changes after NAC. However, that can lead to underestimation of the rCR, which can be mistaken for a NAC effect, which does not happen when a stricter criterion is used.

The variable accuracy of MRI for identifying the various molecular subtypes was analyzed in a previous study involving 264 patients^(^^17^^)^, which found its overall sensitivity and specificity to be 44% and 90%, respectively. However, in triple-negative tumors, not only was the pCR rate higher (46%), but the sensitivity and specificity of MRI also increased significantly, to 60% and 100%, respectively. In the present study, MRI had 83% sensitivity and 74% specificity for that subtype.

Another study investigating factors that contributed to the discrepancy between MRI and the pathology in terms of tumor size found that molecular subtype, nuclear grade, and initial MRI pattern had significant impacts. The discrepancy was lower for the triple-negative subtype than for estrogen receptor-positive tumors^(^^20^^)^.

The results of the present study should be considered in the context of certain limitations. This was a retrospective study, with no specific follow-up, albeit with a posterior analysis of the results. In addition, it was conducted at a single cancer center and did not compare the accuracy of NAC response detection by other imaging methods, such as ultrasound and mammography.

The results presented herein confirm that MRI has a high sensitivity in identifying a pCR after NAC. Its high positive predictive value for the triple-negative and HER2+ subtypes indicates the possibility of using imaging methods for the follow-up of cases that show a complete response on MRI, after histology confirmation of a pCR, as an alternative to surgery. Nevertheless, the lower accuracy of MRI in detecting complete responses in luminal tumors could lead to an underestimation of malignancy and inappropriate breast-conserving surgery.

## CONCLUSION

MRI showed good accuracy in predicting the response to NAC in breast cancer. The sensitivity and positive predictive value of MRI in detecting a pCR were highest for the triple-negative and HER2-enriched subtypes, which are considered the most aggressive.
